# Multiple-Genome-Based Simple Sequence Repeat Is an Efficient and Successful Method in Genotyping and Classifying Different Jujube Germplasm Resources

**DOI:** 10.3390/plants12152885

**Published:** 2023-08-07

**Authors:** Bin Li, Noor Muhammad, Shufeng Zhang, Yunxin Lan, Yihan Yang, Shoukun Han, Mengjun Liu, Meng Yang

**Affiliations:** 1 College of Horticulture, Hebei Agricultural University, Baoding 071000, China; q15731747685@163.com (B.L.);; 2 Research Center of Chinese Jujube, Hebei Agricultural University, Baoding 071000, China

**Keywords:** jujube, Simple Sequence Repeat (SSR), multiple-genome-based SSR (MGB-SSR), genetic diversity, digital ID card

## Abstract

Jujube (*Ziziphus jujuba* Mill.) is a commercially important tree native to China, known for its high nutritional value and widespread distribution, as well as its diverse germplasm resources. Being resilient to harsh climatic conditions, the cultivation of jujube could provide a solution to food insecurity and income for people of arid and semi-arid regions in and outside of China. The evaluation of germplasm resources and genetic diversity in jujube necessitates the use of Simple Sequence Repeat (SSR) markers. SSR markers are highly polymorphic and can be used to evaluate the genetic diversity within and between cultivars of Chinese jujube, and are important for conservation biology, breeding programs, and the discovery of important traits for Chinese jujube improvement in China and abroad. However, traditional methods of SSR development are time-consuming and inadequate to meet the growing research demands. To address this issue, we developed a novel approach called Multiple-Genome-Based SSR identification (MGB-SSR), which utilizes the genomes of three jujube cultivars to rapidly screen for polymorphic SSRs in the jujube genome. Through the screening process, we identified 12 pairs of SSR primers, which were then used to successfully classify 249 jujube genotypes. Based on the genotyping results, a digital ID card was established, enabling the complete identification of all 249 jujube plants. The MGB-SSR approach proved efficient in rapidly detecting polymorphic SSRs within the jujube genome. Notably, this study represents the first successful differentiation of jujube germplasm resources using 12 SSR markers, with 4 markers successfully identifying triploid jujube genotypes. These findings offer valuable information for the classification of Chinese jujube germplasm, thereby providing significant assistance to jujube researchers and breeders in identifying unknown jujube germplasm.

## 1. Introduction

Jujube (*Ziziphus jujuba* Mill.) belongs to the genus *Ziziphus* of family Rhamnaceae [1]. It is an important fruit tree native to China [2,3]. Among the 170 *Ziziphus* species, it has the largest cultivation size and the most commercial and ecological importance [4]. Jujube has been cultivated for over 3000 years. It is highly adaptable and has an abundance of genetic resources [5]. Over 900 types of jujube cultivars necessitate the vital task of examining and classifying jujube germplasm resources. Traditional study and classification approaches in the study of jujube germplasm are mostly based on morphology [6], but they have shortcomings such as low polymorphism, difficulty in identification, and vulnerability to environmental impacts [7].

Similarly, molecular markers used for species classification, such as RAPD, RFLP, AFLP, SRAP, and Simple Sequence Repeat (SSR), offer advantages over morphological classification approaches [8,9,10,11,12]. These markers, having high levels of polymorphism and reproducibility, are evenly distributed in the genome, and are not influenced by factors such as tissue type, developmental stage, or environment [13]. Among them, SSR is the most favored due to its ease of detection, abundant quantity, and high level of polymorphism. This type of marker has been widely used in the assessment of fruit tree germplasm resources and species classification [4,14]. Furthermore, genetic diversity is important in the acquisition, conservation, classification, development, utilization, and improvement of plant germplasm resources [8,15]. Many species including *Z. nummularia* [15], Apricot [16], Taxus [17], olive [18], Jinsha pomelo [19], banana (*Musa* spp.) [20], cotton (*Gossypium* sp.) [21], Grapes [22,23,24], and so on have been subjected to genetic diversity analysis using SSR markers.

SSRs, also known as microsatellites, are short, repeated DNA sequences consisting of 1–6 nucleotide motifs [25]. Identifying core repeat units and designing specific primers are essential for the detection of microsatellite (SSR) molecular markers [1,26]. Traditional SSR screening approaches, such as gene library, interspecies transfer amplification, and public database searches, were time-consuming and inefficient [27,28,29]. Additionally, gel electrophoresis, which is the conventional method to identify SSR typing results, has limitations such as poor resolution and limited capacity for high-throughput screening [30]. Several optimizations have been performed on the SSR assay [31]. For example, the amplification efficiency is improved by optimizing the PCR system [32]; combining multiple SSR markers into one PCR reaction system for multiple PCR can significantly reduce the time and cost of genotyping [33].

The development of SSR markers has become cost-effective now due to reduced sequencing costs and genome assemblies. Based on the genome sequences of three closely related jujube genotypes (‘Dongzao’, ‘Junzao’, and ‘Suanzao’), this study developed an effective SSR screening approach (MGB-SSR) that decreased experimental costs and streamlined the workflow. The 12 SSRs identified by this method effectively discriminate the three jujube genotypes. In addition, the representativeness of these SSRs among different jujube germplasm resources was determined, and based on the results, barcoding IDs for 249 distinct jujubes were generated using the 12 identified SSRs.

## 2. Results

### 2.1. Identification and Screening of SSRs in Three Jujube Genomes

The analysis of SSRs in the three jujube genomes yielded comparable proportions of SSRs with the same core unit length. Trinucleotide repeats constituted the majority, at 71.12% of the total, followed by tetranucleotide repeats at 20.09%, pentanucleotide repeats at 5.53%, and hexanucleotide repeats at approximately 3.25%. Notably, the number of detected SSRs exhibited a positive correlation with genome size (Table 1).

**Figure 1:**
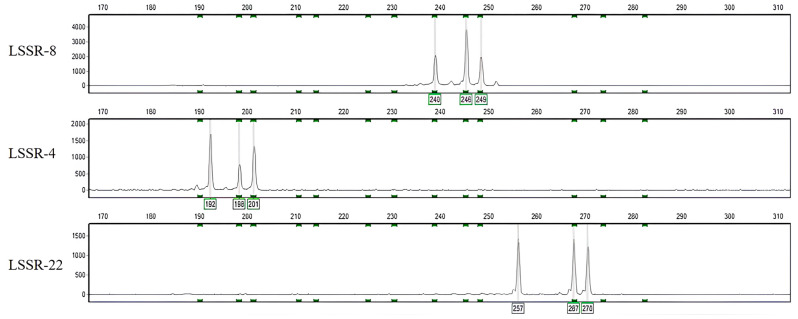
Polyploid amplification results are illustrated as an example. The amplification results from primers LSSR-8, LSSR-4, and LSSR-22 in ‘Zanhuang’ jujube are shown from top to bottom.

By conducting a comparative analysis of the SSR sequences among the three jujube genomes, we identified a total of 286 polymorphic SSR sequences. Initially, sequences that exhibited variations only in two genomes were excluded, followed by filtering based on base mutations, resulting in a final set of 35 SSR sequences (Table S1). Subsequently, we successfully designed 12 pairs of SSR primers that effectively detected the desired target fragments. Notably, LSSR-8, LSSR-4, LSSR-22, and LSSR-25 exhibited successful genotyping of triploid jujube cultivars (refer to Figure 1).

### 2.2. Primer Designing and PCR Amplification of Polymorphic SSRs

Among the 12 pairs of SSR primers that met the criteria, seven contained three-nucleotide repeats, while five contained four-nucleotide repeats. The primers were divided into four groups based on the color of the fluorescent adapter: FAM (green): LSSR-10, LSSR-25, and LSSR-27; HEX (blue): LSSR-17, LSSR-29, and LSSR-26; ROX (red): LSSR-4, LSSR-23, and LSSR-6; TAME (black): LSSR-8, LSSR-28, and LSSR-22 (Table 2).

**Figure 2:**
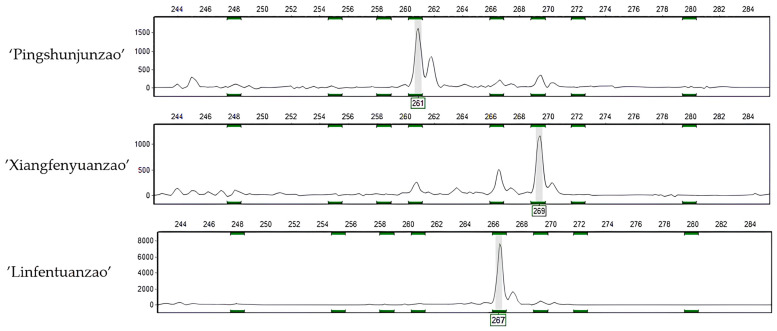
The capillary electrophoresis examination results for three jujube genotypes, namely, ‘Pingshunjunzao’, ‘Xiangfenyuanzao’, and ‘Linfentuanzao’, are shown from top to bottom.

Using these 12 pairs of primers, successful amplification of the expected fragments was achieved for 249 jujubes. Each jujube individual displayed a diverse range of genotyping results. For instance, the LSSR-22 primer revealed three different genotyping results in the samples ‘PingshunJunzao’, ‘Xiangfenyuanzao’, and ‘Linfentuan zao’ (Figure 2).

### 2.3. Population Analysis of Jujube Based on SSR Markers

A total of 106 alleles were identified across 12 polymorphic SSR markers, resulting in an average of 8 alleles per SSR marker. The markers LSSR-22 and LSSR-26 exhibited the highest allelic diversity with 15 alleles, while LSSR-4 and LSSR-6 displayed the lowest diversity with 5 alleles each.

The uniformity of allele frequency was assessed using effective alleles, which ranged from 2.0396 to 7.8924 across all markers, with an average of 3.985 (Table 3). Genetic diversity was measured through expected and observed heterozygosity. The expected heterozygosity ranged from 0.511 (LSSR-4) to 0.875 (LSSR-26), while the observed heterozygosity ranged from 0.485 (LSSR-4) to 0.853 (LSSR-26).

To evaluate the polymorphism within the SSR population, the polymorphism information content (PIC) was calculated, which represents the informativeness of a marker, with higher values indicating greater polymorphism and diversity within the population. The highest PIC value was observed for LSSR-26, reaching 0.861, indicating significant polymorphism within this marker.

**Figure 3:**
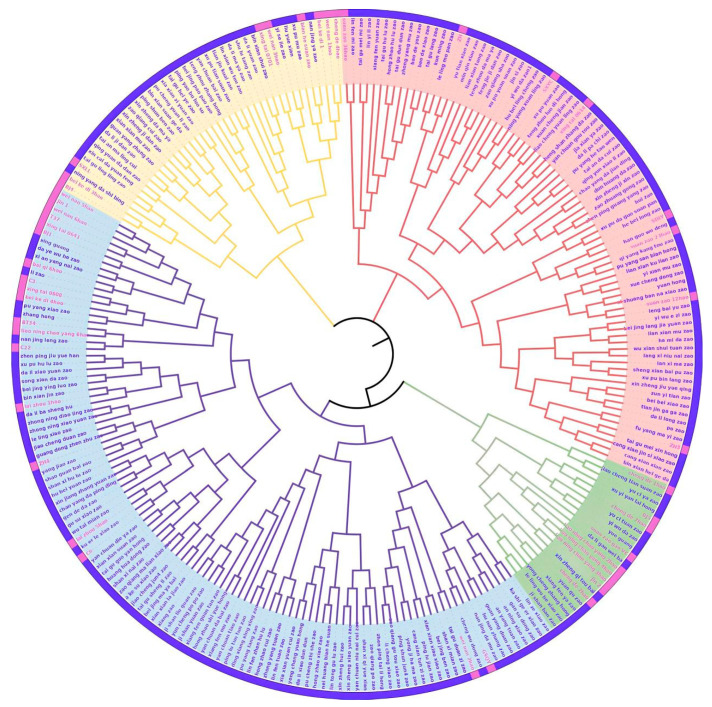
Phylogenetic tree of 249 jujube individuals. The blue, yellow, red, and green branches represent the first, second, third, and fourth groups, respectively. The blue and pink colors in the outside circle are the jujubes and wild jujubes, respectively.

The phylogenetic tree, constructed using SSR data, classified the 249 jujube genotypes into four main groups, with the wild sour jujube dispersed among all groups. The first group comprised the largest number, accounting for approximately 43% (107 jujubes), while the fourth group contained at least 22 genotypes (refer to Figure 3). Jujubes originating from the same geographical region tended to cluster together. For instance, Jujubes such as ‘Liaoningchaoyang2’, ‘Liaoningchaoyang3’, and ‘Liaoningchaoyang4’ were closely positioned within the fourth group, reflecting their geographical proximity.

**Figure 4:**
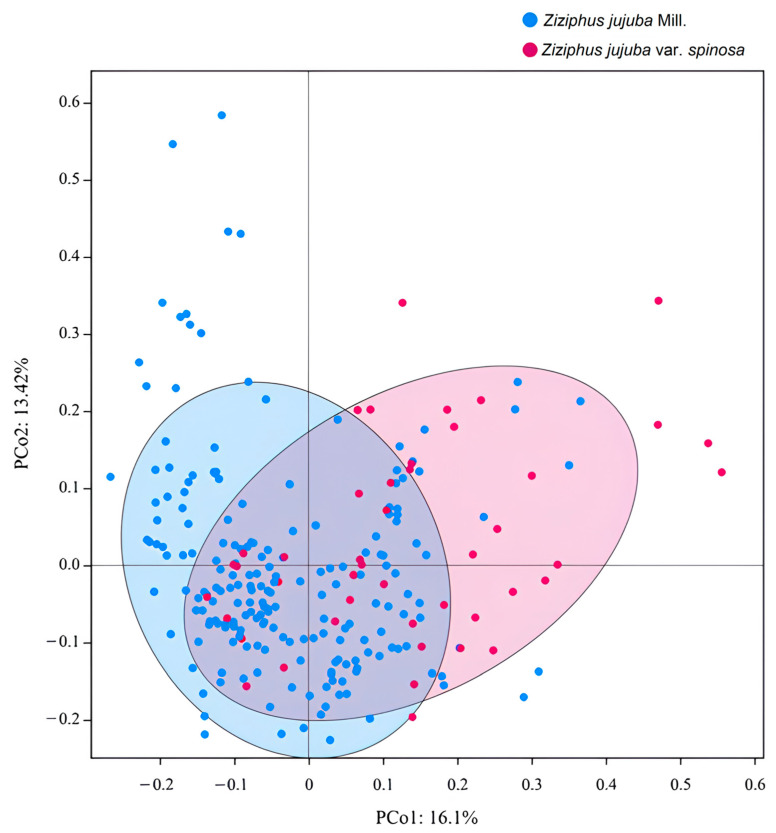
PCoA analysis of 249 jujube individuals. The blue dots represent cultivated jujubes (*Z. jujuba*), while the red dots indicate wild jujubes (*Z. jujuba* var. *spinosa*). Except for a few scattered points, the majority of the dots are evenly distributed, which is consistent with the results obtained from the phylogenetic tree.

Principal coordinates analysis (PCoA) is a useful method for assessing genetic relationships among species. In this study, PCoA was performed on 249 jujube genotypes. By utilizing the first and second principal components as horizontal and vertical coordinates, the jujubes were categorized into two main groups, representing cultivated and wild jujube, respectively. Although there were a few scattered genotypes, the majority of jujube and wild jujube exhibited a close genetic relationship. These findings align with the results obtained from the phylogenetic tree analysis, indicating a high level of genetic similarity between jujube and wild jujube (Figure 4).

### 2.4. Molecular Identity Card of Jujube Varieties

**Figure 5:**
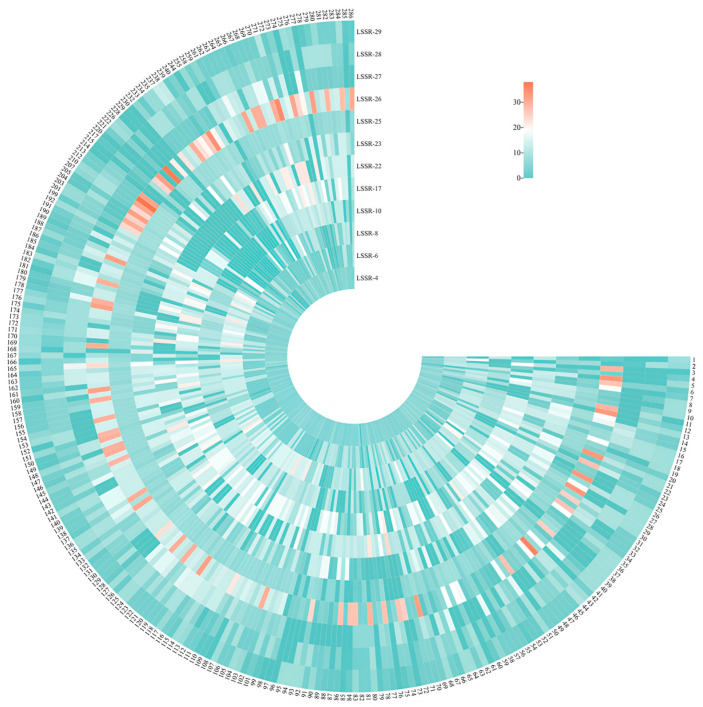
Visualization of the digital ID cards of 249 jujubes. The numbers of corresponding jujubes are provided in Table S2.

The amplification results for all jujubes were organized in a sequential order corresponding to the SSR markers: LSSR-4, LSSR-6, LSSR-8, LSSR-10, LSSR-15, LSSR-17, LSSR-22, LSSR-23, LSSR-26, LSSR-27, LSSR-28, and LSSR-29. This sequential arrangement generated a 24-digit ID for each cultivar (Figure 5 and Supplementary Table S2). Notably, no duplicate numbers were observed between any two different jujubes. This observation indicates that our digital molecular identity numbers can effectively classify individuals within a large jujube population.

## 3. Discussion

The SSR molecular marker technique is widely utilized due to its co-dominant inheritance and high polymorphism. It finds extensive application in genetic diversity analysis, determination of genetic relationships, variety identification, core germplasm selection, and molecular identification [34,35]. Generating SSR-enriched libraries for SSR marker selection is a common approach when limited DNA sequence data are available for a species. However, this method requires significant time and resources due to the need to test polymorphism in a large number of SSR sites from various types. In 2014, SSR molecular markers were first employed in jujube research. A genomic SSR library was generated, and 31 polymorphic SSR markers were used to assess genetic heterogeneity among important jujube cultivars. The results demonstrated high diversity within the jujube population, with average values of Na (5.7), Ne (3.148), Ho (0.678), and He (0.621), surpassing those of other horticultural plants such as apple [36] and peach [37]. According to Botstein et al. [38], SSR markers with a polymorphism information content (PIC) value greater than 0.5 are considered highly polymorphic. In the current analysis, eight out of twelve SSR markers (75%) met this criterion. The average values of Na (8.83), Ne (3.98), Ho (0.62), He (0.70), and PIC (0.65) were comparable to plum [39], orange [40], and pear [41], while exceeding those of mango [42] and wax-apple [43]. Among all the SSR markers, LSSR-26 exhibited the highest values for Na (15), Ho (0.853), He (0.875), and PIC (0.861).

Additionally, based on the detected SSR count and proportion in the three jujube genomes, we observed that the proportion of SSRs with the same core repeat unit length varies only slightly across different genomes (trinucleotide repeats: 71.04–71.29%, tetranucleotide: 19.87–20.19%, pentanucleotide: 5.53–5.58%, hexanucleotide: 3.23–3.25%). Despite being jujube cultivars, ‘Dongzao’, ‘Junzao’, and ‘Suanzao’ exhibit significant genomic differences; for instance, their final assembled genome sizes are 437.7 Mb, 351 Mb, and 406 Mb, respectively. ‘Dongzao’ and ‘Suanzao’ have genome sizes that are more similar to each other compared to ‘Junzao’. According to the statistical analysis of SSR quantity, the genome size, and SSR count show a positive correlation (‘Dongzao’: 455,654 SSR loci; ‘Junzao’: 455,654 SSR loci; ‘Suanzao’: 506,730 SSR loci). In conclusion, SSRs are uniformly distributed in the jujube genome, and the genome size significantly influences the quantity of SSRs.

Traditional methods for developing SSR markers can be classified into three categories: (1) database and literature searches to identify relevant SSR information, (2) cross-amplification using common primers among closely related species, and (3) utilizing software to identify microsatellite loci in genomic DNA (gDNA), complementary DNA (cDNA), and expressed sequence tag (EST) sequences. With advancements in sequencing technology, new methods for identifying SSRs have emerged [44,45,46]. For instance, Li et al. developed a high-throughput SSR genotyping method called AmpSeq-SSR, which combines multiplex PCR and targeted deep sequencing. This method enables the accurate genotyping of thousands of SSRs with over 94% accuracy [26]. Additionally, Tian et al. developed software based on RNA-seq data to identify polymorphic SSRs, and more than 92% of the identified SSRs exhibited polymorphisms [31]. Current methods for optimizing SSR markers primarily focus on the integration of sequencing and genotyping technologies. These approaches can identify a large number of potential SSRs, but determining their polymorphic nature within a population requires a substantial number of PCR experiments, leading to significant time and financial costs.

The MGB-SSR method proposed in this study offers several advantages over traditional methods:

(1) Efficient SSR screening: The utilization of multiple closely related genomes in the MGB-SSR approach allows for the identification and elimination of invalid sites that are identical across genomes, and this avoids the massive selection from thousands of candidate SRRs. This significantly reduces the time and financial resources required for screening.
(2) Enhanced accuracy with capillary electrophoresis: By combining SSR screening with capillary electrophoresis, the MGB-SSR method overcomes the limitations associated with traditional gel electrophoresis. Issues such as uneven distribution of PCR products between lanes due to variations in gel concentration and voltage are eliminated, resulting in more accurate and reliable test results.
(3) Universal applicability: With advancements in sequencing technology, it has become feasible to obtain 2–3 closely related genomes for most fruit trees. This means that the SSR markers developed using MGB-SSR can be applied across various species within the same family or even different genera. Furthermore, genetic diversity analysis has demonstrated that the SSR markers developed through MGB-SSR exhibit significant polymorphism, as indicated by the Na, Ne, and PIC parameters meeting the standard criteria.
(4) Detectable polyploidy: The MGB-SSR method has shown effectiveness in detecting polyploid jujube germplasm resources. With the increasing number of jujube hybrid varieties, the evaluation and identification of polyploid jujube are becoming more important. In this study, four of the SSR markers utilized effectively characterized polyploidy in jujube, demonstrating their potential for this application.
(5) Compared to utilizing markers from the SNP array and the commonly used WGS strategy, which require a substantial amount of genome re-sequencing for genotyping and classifying different jujube germplasm resources, our method using only 12 SSRs significantly reduces both time and financial costs.

In summary, the MGB-SSR method provides a more efficient and accurate approach to SSR screening, offering cost and time savings, improved accuracy through capillary electrophoresis, and broad applicability for genetic diversity analysis in fruit trees and other related species. Additionally, it shows promise in detecting polyploidy in jujube germplasm resources.

The utilization of a set of highly polymorphic SSR markers significantly reduces the possibility of concordance between individuals, ensuring accurate molecular identification. This technology has been successfully applied in various crops such as nuts and melons [47,48,49,50]. Given the vast number of jujube varieties, with over 900 currently available, investigating and classifying jujube germplasm resources becomes a challenging task. The presence of homonyms and different names further complicates the situation, leading to mixed varieties and subpar products in the market. Therefore, the establishment of digital ID cards is crucial for standardized jujube plant management. In this study, 12 pairs of SSR primers were specifically designed and employed to create exclusive molecular markers for the 249 jujube plants analyzed. This comprehensive approach enabled the complete differentiation of all 249 jujube varieties. These findings provide a valuable reference for the identification and classification of jujube plant varieties. The development of digital ID cards based on SSR markers offers a reliable and efficient approach to authenticate and differentiate jujube plants, contributing to improved management practices and ensuring the authenticity and quality of jujube products in the market.

## 4. Materials and Methods

### 4.1. Availability of the Jujube Genomic Data

The genome data of three jujube genotypes were downloaded from NCBI’s Genome database (www.ncbi.nlm.nih.gov, accessed on 11 January 2022.): ‘Dongzao’ genome [51] (JREP00000000); ‘Junzao’ genome [52] (LPXJ00000000); ‘Suanzao’ genome [53] (JAEACU010000000).

### 4.2. Plant Material and DNA Extraction

Fresh green leaves of the adult trees were collected from 249 jujube genotypes and placed in a −80 °C freezer for storage (Table S2). A modified Cetyltriethylammnonium Bromide (CTAB) method [54] was used to extract genomic DNA from jujube leaves for each sample. The purity of DNA was verified using a NanoDrop One UV-Vis spectrophotometer (Thermo Fisher Scientific, Waltham, MA, USA), and the integrity of DNA was assessed by agarose gel electrophoresis (Table S2).

### 4.3. Preliminary SSR Identification of Jujube Genome

The MISA program [55] (version 1.0, http://pgrc.ipk-gatersleben.de/misa/misa_html, accessed on 18 March 2022.) was used to identify the SSR loci in the three jujube genomes, and the parameters were set to 3–6 nucleotides for the core repeat unit and the number of repeat times <3. Subsequently, the core unit length, number of repetitions, and repetition frequency were counted, and the distribution properties of SSR in the jujube genome were studied by comparing the identified results of the three genomes.

### 4.4. SSR Screening of Three Jujube Cultivar Genome Polymorphisms

Based on the SSR position information obtained from the MISA program, we extracted the core repeat unit and 300 bp conserved sequences at both ends of each SSR from the genome. Using these extracted SSR sequences, we then performed a screening process to identify the polymorphic SSRs in the three genomes. The steps for this screening process were as follows:

(1) Create Blast databases for the three jujube genomes.
(2) The SSR sequences were subjected to Blast alignment against the corresponding jujube genome database, and subsequently, non-unique results were removed, retaining only the specific SSRs.
(3) The remaining specific SSRs were subjected to a mutual comparison with the other two jujube genome databases. During this comparison, any results that were found to be identical within any of the two jujube genomes were discarded. Additionally, SSRs showing less than 90% consistency across the three genomes were also excluded.
(4) All the remaining results were statistically merged, and those with consistent conservative sequences at both ends but differing core repeat units were selected.
(5) Eliminate the results that show differences only between two genomes, thus highlighting the variations among the three genomes at the SSR level.

**Figure 6:**
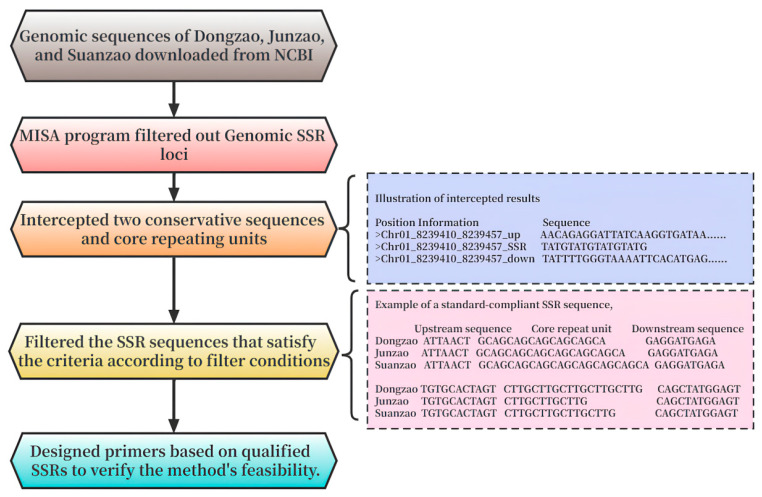
Flowchart of the MGB-SSR method.

The pipeline is displayed in Figure 6.

### 4.5. Design and Detection of Polymorphic SSR Primers

The selection of primers for SSRs followed specific criteria using the Primer Premier 6 software (version 6.24) [56]. The chosen primers met the following conditions:

(1) They were designed in conserved regions near both ends of the core repeat sequences;
(2) The length of the amplicons ranged between 50 and 300 bp;
(3) The primers had similar annealing temperatures;
(4) To prevent primer dimers, there was no complementary sequence between the primers;
(5) Primer specificity was assessed using NCBI-BLAST;
(6) The upstream primer had an 18 bp M13 linker sequence added to the 5′ end, which matched with fluorescent linker primers of different colors (FAM blue, HEX green, ROX red, and TAME black).

### 4.6. Primer Performance Evaluation

To evaluate the efficacy of each primer pair, 6-FAM fluorescent adapter primers were employed, and test samples were selected from a diverse range of jujube varieties collected from different locations in China. The sample set consisted of 26 diploid jujubes (numbered 1–26) and 4 triploid jujube cultivars, including ‘Zanhuangdazao’ and 3 triploid progenies resulting from the cross between ‘Dongzao’ and ‘Chenguang’. This selection ensured the representation of a wide spectrum of SSR markers. The PCR amplification results were observed through on-machine detection to assess the characteristics of the samples.

### 4.7. PCR Amplification System

The PCR (polymerase chain reaction) system was carried out in a volume of 20 μL: 2× Taq PCR buffer 10 μL; 10× primer 0.6 μL (upstream primer 0.1 μL, downstream primer 0.3 μL, fluorescent linker primer 0.2 μL); genomic DNA 20 ng with ddH20 to 20 μL. The temperature profile was set as 95 °C (5 min); cycles, 20 times (95 °C/30 s; 52 °C/30 s, 72 °C/30 s, 95 °C/30 s, 50 °C/30 s, and 72 °C/30 s); 72 °C (10 min) for final extension. Then, the product was stored at 4 °C.

### 4.8. PCR Product Detection by Capillary Electrophoresis

Following the detection of the PCR product through agarose electrophoresis, 0.3 μL of the PCR product, 0.5 μL of the molecular weight internal standard, and 9.5 μL of deionized formamide were combined and added to the PCR plate. The mixture was then denatured at 95 °C for 5 min, cooled at 4 °C, and centrifuged. For machine detection, 1× buffer was added. The detection findings were entered into Genemarker (version 2.2.0), and subsequently, the electropherogram and site information table were exported.

### 4.9. Data Analysis and Application

Using the aforementioned system, PCR amplification was performed on all DNA samples, and the results were analyzed. The software Popgen 32 (version 1.32) was utilized to calculate various parameters such as the number of alleles, effective number of alleles, and expected heterozygosity for each sample. The SSR data were then converted into a 0/1 matrix (representing the presence or absence of stripes) and analyzed using the ‘ape’ package (version 5.7-1) in R language [57]. The evolutionary tree of the system was constructed using the ‘ggtree’ package (version 3.17) [58,59].

The genotyping results for each SSR marker were sorted and assigned numbers. The value ‘00’ indicated undetectable or missing typing, while the remaining values ranged from ‘01’ to ‘99’, ordered in ascending order (Supplemental Table S2). Subsequently, corresponding to each jujube, a sequential digital ID card for jujubes was generated in series [60]. Finally, the Chi Plot (www.chiplot.online, accessed on 6 February 2023.) tool was used to visualize the generated ID cards.

## 5. Conclusions

This study presents a novel method, MGB-SSR, for identifying SSRs in the jujube genome. MGB-SSR offers a rapid and cost-effective approach to screening polymorphic SSR markers with superior efficiency and affordability in comparison with the existing methods. Initially, we individually identified SSR loci in the three jujube genomes. Notably, the number of SSRs in these genomes displayed a positive correlation with their respective genome sizes, while the proportion of SSRs with the same core unit length remained consistent across different jujube genomes. Subsequently, we eliminated duplicate SSRs within the three jujube genomes, yielding a total of 286 SSR loci, with 35 of them exhibiting differences among the three jujube varieties. Through successful primer design, we targeted 12 of these polymorphic SSRs, enabling the generation of digital ID cards for jujube plants. This groundbreaking effort resulted in the successful differentiation of 249 distinct jujube genotypes/germplasms, offering valuable insights for the classification of jujube germplasm resources. Additionally, four of the SSR markers proved effective in detecting triploid jujube genotyping, providing reference data and methodological support for the development of polyploid jujube SSR markers. Furthermore, since MGB-SSR relies on comparative analysis among closely related genomes, it holds the potential for extension to other species. Overall, this study introduces an advantageous and innovative approach to screening polymorphic SSR markers, highlighting the merits of the MGB-SSR method.

## Figures and Tables

**Table 1:** Distribution and frequency of SSRs in three jujube genomes.

Core Sequence (bp)	3	4	5	6	Total
‘Dongzao’ (437.7 Mb)	361,995 (71.12%)	102,275 (20.09%)	28,154 (5.53%)	16,542 (3.25%)	508,966 (100%)
‘JunZao’ (351 Mb)	324,838 (71.29%)	90,541 (19.87%)	25,444 (5.58%)	14,831 (3.25%)	455,654 (100%)
‘SuanZao’ (406 Mb)	359,973 (71.04%)	102,309 (20.19%)	28,076 (5.54%)	16,372 (3.23%)	506,730 (100%)

**Table 2:** Overview of the expected sizes, core units, and fluorescent labels of the 12 SSRs.

SSR Marker	Lengths	Primer Orientation	Primer Sequence 5′-3′	Core Unit	Dye
LSSR-4	209	Reverse	ATGCTGCCAGGAGTGTTCAATA	(GCA)7	ROX
		Forward	GCCTTCGTCTAATTCCTCTCTGAT		
LSSR-6	309	Reverse	GCTCTATTTCTCTACCATTCTCACACT	(CAT)4	ROX
		Forward	CATTCAGCATCAACAATATCCTCCA		
LSSR-8	252	Reverse	CCATTGGTAACAGCAAGTT	(GAA)6	TAME
		Forward	TAGTCTCTTCTCTGGCTATAC		
LSSR-10	129	Reverse	GAAAGCCATAACTCGTTGATCTTGT	(CTTG)5	FAM
		Forward	GCTCGCCACATAACAGGATACA		
LSSR-17	141	Reverse	CAAGAAGATACAAACCCACCAATCA	(GACA)4	HEX
		Forward	TGGAGGACTGTTCCTACCAATAC		
LSSR-22	267	Reverse	AACAGACATGGCTATGGTGGAATT	(TTA)6	TAME
		Forward	CAAAGACCGAAAGAAAGTTCAGCAA		
LSSR-23	217	Reverse	ATGAAGTCGTCGCTGTCAAGTG	(TAT)4	ROX
		Forward	CAAGATCCAGCCAAAGTCAAAGTTT		
LSSR-25	121	Reverse	CCAGAACTACTCAGAACTTCTATCATC	(AAT)4	FAM
		Forward	TAGCGTTTGCAGGTTGCTTAGT		
LSSR-26	172	Reverse	GGAAGGACTTTGTCAGCATGGTAG	(GTT)12	HEX
		Forward	AACAGCATATTTGGATCCATTTCG		
LSSR-27	136	Reverse	CACTGCAAATGCTTTGTCATCTTT	(TATG)6	FAM
		Forward	AAAGCATCACCCATCCTCTACATC		
LSSR-28	257	Reverse	CGTGGACCAAGTCTATACCAAAATG	(ATA)9	TAME
		Forward	TGGTTTTTCTTCTCCTAATCCATGTG		
LSSR-29	145	Reverse	TCAATAATTCCAGCCGAATCCTTA	(TATA)5	HEX
		Forward	TGGGAGTCTAGCTTCATTCAAACA		

**Table 3:** Population characteristics of SSR markers based on 249 jujubes.

SSR Marker	Na	Ne	HObs	HExp	PIC
LSSR-4	5	2.0396	0.485	0.511	0.431
LSSR-6	5	2.922	0.845	0.659	0.598
LSSR-8	11	6.1057	0.732	0.839	0.818
LSSR-10	10	2.994	0.576	0.68	0.624
LSSR-17	9	2.919	0.604	0.663	0.633
LSSR-22	15	6.198	0.551	0.841	0.823
LSSR-23	9	5.2615	0.752	0.812	0.783
LSSR-25	6	2.2674	0.461	0.56	0.461
LSSR-26	15	7.8924	0.853	0.875	0.861
LSSR-27	9	4.3823	0.506	0.786	0.751
LSSR-28	6	2.1094	0.444	0.527	0.426
LSSR-29	6	2.7283	0.646	0.635	0.564

Note: Na = total number of observed alleles; Ne = effective number of alleles; HObs = observed heterozygosity; HExp = expected heterozygosity; PIC = polymorphic information content.

## Data Availability

All data in this study can be found in the manuscript or supplementary materials.

## Supplementary Materials

The following supporting information can be downloaded at: https://www.mdpi.com/article/10.3390/plants12152885/s1, Table S1: The information of 35 candidated SSRs; Table S2: The digital ID card of 249 jujube genotypes.
